# Tea Saponin Exerts Dose-Dependent Dual Effects on Growth and Hepatic Health in Hybrid Grouper (*Epinephelus fuscoguttatus* ♀ × *E. lanceolatus* ♂) Fed a High-Lipid, Low-Protein Diet via Redox-Immune Regulation

**DOI:** 10.3390/ani16091408

**Published:** 2026-05-04

**Authors:** Shengrong Guo, Jun Yu, Lili Shi, Beiping Tan, Shuang Zhang, Xiaobo Yan

**Affiliations:** 1 Laboratory of Aquatic Nutrition and Feed, College of Fisheries, Guangdong Ocean University, Zhanjiang 524088, China; gsr02112112@163.com (S.G.);; 2 Ocean College, Fujian Polytechnic Normal University, Fuqing 350300, China; 3 Department of Marine Biology, Shenzhen Institute of Guangdong Ocean University, Shenzhen 518116, China; 4 Aquatic Animals Precision Nutrition and High Efficiency Feed Engineering Research Center of Guangdong Province, Zhanjiang 524088, China

**Keywords:** tea saponin, oxidative stress, immune homeostasis, inflammatory cytokines, transcriptomics

## Abstract

High-lipid, low-protein diets are economically advantageous in aquaculture costs but often induce hepatic damage, immunosuppression and metabolic disorders in carnivorous fish like hybrid grouper (*Epinephelus fuscoguttatus* ♀ × *E. lanceolatus* ♂). Tea saponin (TS) is a bioactive triterpenoid from camellia seed meal with excellent lipid-lowering and anti-inflammatory effects. The purpose of the current study was to investigate the dose-dependent impacts of TS supplementation at 0% (control, T0), 0.05% (T5), 0.10% (T10), 0.15% (T15), and 0.20% (T20) in diets formulated to contain 42% crude protein and 16% crude lipid on juvenile hybrid grouper (17.51 ± 0.03 g), with three replicate tanks per treatment, over a 4-week feeding trial. The results showed that low TS supplementation (0.05%) improved growth, with the effect limited to final average weight (FAW), whereas higher doses (≥0.15%) reduced weight gain. The T5 group exhibited the highest hepatic total antioxidant capacity and superoxide dismutase activity, consistent with the corresponding gene expression, and the lowest malondialdehyde and reactive oxygen species levels. Lysozyme activity and immunoglobulin M content were significantly higher in T5–T15 groups (*p* < 0.05). Furthermore, appropriate TS significantly upregulated anti-inflammatory cytokines (*p* < 0.05) and downregulated pro-inflammatory factors. Histologically, 0.10% TS reduced inflammatory infiltration, while high doses caused hepatocyte rupture and lipid vacuolation. Transcriptomic analysis further elucidated that the beneficial effects of low-dose TS were linked to the activation of PPAR signaling, fatty acid catabolism, and cellular quality control pathways, while high-dose TS triggered stress-related and biosynthetic programs. In conclusion, moderate TS supplementation (0.05–0.10%) ameliorated diet-induced oxidative stress and immunosuppression, improved growth performance and anti-inflammatory factor expression, whereas over-addition inhibited growth and exacerbated hepatic damage. Based on a quadratic model, the optimal dietary TS level for maximizing growth under this low-protein, high-lipid regimen is estimated at 0.055%.

## Simple Summary

High-lipid, low-protein feeds can help to reduce aquaculture costs and lower nitrogen waste, but they may also damage liver health and weaken disease resistance in hybrid grouper, a high-value marine fish widely farmed in Asia. In this study, we examined whether tea saponin, a natural compound from camellia seed meal, could help to protect these fish when they were fed this kind of diet. We found that adding 0.05–0.10% tea saponin improved growth, supported liver health, strengthened the body’s natural ability to fight oxidative cell damage, and reduced inflammatory cell infiltration and pro-inflammatory cytokine expression. However, too much tea saponin had the opposite effect, slowing growth and causing clear signs of liver injury. The best results were seen at a relatively low inclusion level, showing that careful dose control is essential. These findings suggest that tea saponin could be developed as a practical feed additive to improve fish health and production efficiency when high-lipid, low-protein feeds are used. This could help farmers to reduce feed costs and ensure more reliable production of this valuable marine fish.

## 1. Introduction

Lipids are indispensable for fish growth and various physiological activities, including reproduction and migration. Although teleost fish, particularly carnivorous marine species, have traditionally been considered inefficient at utilizing carbohydrates [1], recent studies have shown that these species retain some capacity to metabolize carbohydrates when dietary inclusion levels are moderate and the carbohydrate sources are digestible [2]. Nevertheless, long-term intake of high-carbohydrate diets can still lead to glucose metabolism disorders [3], growth impairment, and various pathological conditions in carnivorous species [4,5]. For this reason, lipids are frequently considered a major energy source for carnivorous fish [6]. As one of the macronutrients in daily diets, lipids are absorbed and digested in the intestines to release fatty acids, which are then transported via the bloodstream to peripheral tissues. Some of these fatty acids are stored or oxidized to provide metabolic energy, while others contribute to maintaining the structure and function of cell membranes [7]. Additionally, lipids play a critical role in the development of nervous tissues such as the eyes and brain in fish. Moreover, dietary lipids can directly or indirectly modulate the activity of various transcription factors involved in maintaining lipid homeostasis [8].

Given these physiological roles, dietary lipids have become increasingly important in modern aquafeed formulation. Advances in nutritional physiology, aquaculture technology, and economic constraints have driven the industry toward more cost-effective feed formulations, often with higher lipid content. This is particularly relevant in the farming of carnivorous fish, where the protein sparing effect can be utilized to reduce the use of expensive protein ingredients [9]. Moreover, such feed formulations help to decrease the discharge of nitrogenous waste into water bodies during the farming process. This contributes to environmentally sustainable production and helps to conserve water resources [10], which are essential for human survival.

Accordingly, high-lipid and low-protein diets have been increasingly adopted in aquaculture because of their economic and potential environmental benefits. Chen et al. [11] utilized a high-lipid, low-protein regimen for raising triploid rainbow trout (*Oncorhynchus mykiss*) after evaluating its positive effects on growth performance. Extensive research supports the feasibility of moderately reducing dietary protein, as it shows no significant negative impact on weight gain, specific growth rate, feed conversion ratio, or certain antioxidant indicators in Nile tilapia (*Oreochromis niloticus*) [12] and Gibel carp (*Carassius auratus gibelio*) [13]. Similarly, a moderate increase in dietary lipid does not necessarily impair key production traits. For example, increasing dietary lipid from 2.7% to 18.1% did not significantly affect survival in large yellow croaker (*Larimichthys croceus*) [14], whereas increasing dietary lipid from 14% to 25% in juvenile Atlantic halibut (*Hippoglossus hippoglossus*) did not significantly affect growth, feed conversion ratio, or muscle lipid content, although whole-body and liver lipid deposition increased [15]. Beyond these neutral effects, such diets can enhance nutritional efficiency, markedly improving protein or nitrogen retention in white seabass (*Atractoscion nobilis*) [16], hybrid grouper (*Epinephelus fuscoguttatus* ♀ × *E. lanceolatus* ♂) [6], and increasing the protein efficiency ratio in largemouth bass (*Micropterus salmoides*) [17].

However, the benefits of such diets are limited, as excessive lipid intake can lead to adverse health effects, including fatty liver, obesity, and other lipid metabolism disorders [18]. These effects are often linked to cellular stress pathways. At the intestinal level, a diet with 18.65% lipid (high-fat diet) induced endoplasmic reticulum stress and impaired antioxidant capacity in largemouth bass (*Micropterus salmoides*) [19]. Feeding high-lipid diets has been shown to cause oxidative stress and apoptosis in various fish species, such as tilapia (*Oreochromis niloticus*) [20], yellow catfish (*Pelteobagrus fulvidraco*) [21], and grouper (*Epinephelus fuscoguttatus* ♀ × *E. lanceolatus* ♂) [22]. Furthermore, specific molecular pathways can be activated by dietary lipids; for example, a diet containing 18% lipids significantly increased the phosphorylation level of c-Jun N-terminal kinase (JNK) and Mitogen-activated protein kinase (MAPK) pathway in the head kidney of turbot (*Scophthalmus maximus* L.) [23]. In addition to the effects of high fat, a deficiency in dietary protein is also detrimental. In tilapia (*Oreochromis niloticus*), insufficient protein intake has been reported to decrease the white blood cell count [24] and suppress lysozyme activity [25].

These risks highlight the need for effective nutritional interventions. Tea saponin (TS) is a bioactive mixture of pentacyclic triterpenoid saponins with similar structure and is widely distributed in *Camellia oleifera* seed meal. As natural glycosides, TS offers advantages of ready availability and cost-effectiveness, and its extraction and purification methods have garnered increasing research interest [26]. TS exhibits pharmacological effects at relatively low and safe doses. It is considered to have a high safety profile for mammals because it cannot permeate the intestinal wall to be absorbed into the bloodstream [27]. In murine studies, TS can ameliorate immune imbalance, repair intestinal barrier damage, and alleviate gut dysbiosis [28]. Furthermore, its efficacy in enhancing immune performance has been confirmed in poultry and ruminants [29,30]. However, the potential nutritional and immunomodulatory benefits of TS remain unexplored in marine fish.

To address this gap, we focused on hybrid grouper (*Epinephelus fuscoguttatus* ♀ × *E. lanceolatus* ♂), an economically important carnivorous marine fish in Asia. It is valued for its rich nutritional quality and rapid growth performance, which have supported its expanding aquaculture production [31]. However, the rising cost of high-protein feeds poses a significant challenge to its sustainable farming. Our previous studies indicated that while high-lipid diets (16%) or low-protein, high-lipid diets (42% protein, 16% lipid) did not significantly compromise the growth performance of hybrid grouper, they induced adverse health effects, including immune suppression, hepatic damage, inflammation and lipid accumulation [32,33]. These findings highlight an urgent need for a widely available, green, and efficient feed additive to alleviate these negative symptoms. Given its documented antioxidant, anti-inflammatory and lipid-lowering properties, TS represents a promising candidate for this purpose. Therefore, this study aimed to investigate the effects of dietary TS supplementation on the growth, immunity, and hepatic health of hybrid grouper fed a low-protein, high-lipid diets, thereby providing a theoretical basis for the application of TS in aquafeeds.

## 2. Materials and Methods

### 2.1. Ethics Statement

All fish used in the experiments were handled in accordance with the scientific protocols established by the Animal Ethics Committee of Guangdong Ocean University (Approval number: GDOUIACUC-2024-A0108).

### 2.2. Experimental Diets

The ingredients and nutrient composition of the experimental feeds are shown in Table 1. Based on our previous study [32,33], the present experimental feeds were formulated as low-protein, high-lipid diets suitable for hybrid groupers, with dietary protein and lipid levels set at 42% and 16%, respectively, while meeting the basic nutritional requirements. Five graded levels of TS were established: 0, 0.5, 1.0, 1.5, and 2.0 g/kg diet (equivalent to 0%, 0.05%, 0.10%, 0.15%, and 0.20%), designated as T0, T5, T10, T15, and T20, respectively. The TS with a purity of 96.26% was purchased from Hanqing Biotechnology Co. (Chenxi, China). The diets was prepared as follows: First, all the ingredients were sieved through a 60-mesh sieve. Then, all the components were precisely weighed according to the formulation and thoroughly mixed. The mixture was subsequently processed using a twin-screw extruder at room temperature with a screw speed of 500–600 rpm to produce pellets with a diameter of 2.5 mm. The pellets were then dried at ambient temperature until the moisture content reached approximately 10%. Finally, the finished diets were sealed and stored at −20 °C, first used within 1 week after preparation, and removed in portions as needed throughout the feeding trial.

### 2.3. Fish and Feeding Trial

Juvenile hybrid grouper were obtained from a local farm on Donghai Island (Zhanjiang, China). Prior to the experiment, they were acclimatized to the local environment in concrete tanks (5 m× 4 m× 1.8 m) at the Marine Biology Research Base of the Guangdong Ocean University (Zhanjiang, China). During this 14-day acclimation period, the fish were fed a commercial diet containing 46% crude protein and 12% crude lipid. Subsequently, 450 hybrid grouper fish of uniform size (initial weight: 17.51 ± 0.03 g) and with intact body surfaces were selected and randomly distributed into 15 opaque fiberglass tanks (500 L each), resulting in 30 fish per tank. Each of the five experimental dietary treatments was assigned to three replicate tanks in a completely randomized design. As this study was designed as a “metabolic challenge” rather than a long-term growth trial, the feeding experiment lasted for 4 weeks, which was sufficient to induce metabolic stress and assess the protective effects of TS [34,35,36]. The fish were fed their respective experimental diets to apparent satiation twice daily at 08:00 and 17:00. Feed intake was recorded daily. To maintain stable water quality, approximately 80% of the water in each tank was replaced daily. During the feeding trial, water temperature was maintained at 28–32 °C, salinity at 25–30‰, pH at 8.0–8.2, and dissolved oxygen at above 5.0 mg/L. Fish were reared under the natural photoperiod.

### 2.4. Sampling

Following a 24 h fasting period, all fish were sampled at the end of the feeding trial. First, the total weight and number of fish in each tank were recorded to calculate growth performance parameters. Six fish were then randomly selected from each tank and anesthetized with clove oil before sampling, and approximately 0.8 mL of blood per fish was collected from the tail vein into 1.5 mL centrifuge tubes using a sterile 1 mL syringe. After clotting at 4 °C for 12 h, the samples were centrifuged at 4 °C and 4000 rpm for 15 min, and the obtained serum was stored in a −80 °C refrigerator for analysis of serum biochemical indices. Liver and intestinal samples were collected from three fish after blood sampling, these tissues were snap-frozen in liquid nitrogen and then stored at −80 °C for later determination of enzyme activities and gene expression. For histological observations, two additional fish per tank were randomly selected, their livers and intestines were dissected, fixed in 4% formaldehyde solution, and kept for morphological analysis. To assess the intestinal microbiota, three fish per tank were aseptically dissected using sterile instruments. The entire intestines, along with its contents were placed into RNA-free tubes, and quickly frozen at −80 °C for downstream microbial analysis.

### 2.5. Analysis of Basic Dietary Components

The contents of crude protein, crude lipid, and moisture in feed were determined according to AOAC methods [37], using the Kjeldahl, Soxhlet extraction, and oven-drying methods, respectively.

### 2.6. Histological Analysis of Liver

Liver tissue samples were fixed in 4% neutral buffered formaldehyde for 24 h, dehydrated through a graded ethanol series, cleared in xylene, and embedded in paraffin wax. Serial sections (5 μm thick) were cut using a microtome, mounted onto glass slides, and subjected to deparaffinization in xylene followed by rehydration through a descending ethanol series. The sections were then stained with hematoxylin for 8 min and eosin for 1 min at room temperature, and examined under a light microscope (ECLIPSE Ni-E, Nikon, Tokyo, Japan). Representative images were captured for histopathological evaluation.

### 2.7. Analysis of Immunological and Biochemical Parameters in Liver Samples

Total protein (TP; No. ml095441) concentration, immunological and biochemical parameters, including superoxide dismutase (SOD; No. ml926247), total antioxidant capacity (T-AOC; No. ml093084), glutathione reductase (GR; No. ml016834), reactive oxygen species (ROS; No. ml955621), malondialdehyde (MDA; No. ml555268), immunoglobulin M (IgM; No. ml326413), lysozyme (LYZ; No. ml556394), acid phosphatase (ACP; No. ml445860), and alkaline phosphatase (ALP; No. ml555961) were measured in liver tissue homogenates using commercial kits from Enzymelinked Biotechnology Co., Ltd. (Shanghai, China). Liver tissue blocks were rinsed with ice-cold saline to remove surface contaminants, accurately weighed, and homogenized in ice-cold saline at a ratio of 1 g tissue to 5 mL saline using a tissue grinder at 4 °C. The homogenates were centrifuged at 2500× *g* for 10 min at 4 °C, and the resulting supernatants were collected, aliquoted, and stored at −80 °C until analysis. Before assay, supernatants were diluted as needed, and all assays were performed according to the manufacturer’s instructions.

### 2.8. Liver RNA Extraction and Quantitative Real-Time PCR

Approximately 90 mg of liver tissue per sample was cryogenically ground in liquid nitrogen. Total RNA was extracted using the TransZol Up Plus RNA Kit (Cat. No.: ER501, TransGen Biotech Co., Ltd., Beijing, China). RNA integrity was assessed by formaldehyde denaturing agarose gel electrophoresis, while RNA purity and concentration were determined using a NanoDrop 2000 spectrophotometer (Thermo Fisher Scientific Inc., Waltham, MA, USA). Samples with clear 28S and 18S rRNA bands and an OD_260_/OD_280_ ratio between 1.8 and 2.0 were used for cDNA synthesis. High-quality RNA was reverse-transcribed into cDNA using a gDNA-removing reverse transcription premix (RT101-01, Vazyme Biotech Co., Ltd., Nanjing, China). Quantitative real-time PCR (RT-qPCR) was performed on a LightCycler^®^ 480 II system (F. Hoffmann-La Roche AG, Basel, Switzerland) equipped with a 384-well plate module, using SYBR qPCR Master Mix (Q312-02, Vazyme Biotech Co., Ltd.) according to the manufacturer’s protocol. Gene-specific primers (listed in Table 2) were synthesized by Sangon Biotech Shanghai Co., Ltd. (Shanghai, China), and *β-actin* was used as the reference gene. The thermal cycling conditions were as follows: initial denaturation at 95 °C for 30 s, followed by 40 cycles of denaturation at 95 °C for 10 s, annealing at 56 °C for 30 s, and extension at 72 °C for 30 s. The amplification efficiency of all primer pairs was evaluated using a standard curve generated from serial dilutions of cDNA, and the primer efficiencies ranged from 92% to 104%. Relative mRNA expression levels were calculated using the 2^−ΔΔCT^ method [38].

### 2.9. Transcriptome Sequencing and Analysis

Based on the growth performance and liver histomorphology results, three groups (T0, T5, and T20) were selected for transcriptome sequencing, with three biological replicates in each group. Prior to library construction, RNA integrity was rigorously scrutinized using the RNA Nano 6000 Assay Kit on the Agilent Bioanalyzer 2100 system (Agilent Technologies, Santa Clara, CA, USA) to ensure optimal sample quality. Following the manufacturer’s protocols, cDNA libraries were constructed and subjected to high-throughput sequencing on the Illumina NovaSeq platform, generating 150 bp paired-end reads. For raw data processing, clean reads were obtained by filtering low-quality sequences and adapters. These high-quality reads were then mapped to the reference genome utilizing the HISAT2 software (version 2.0.4). Differentially expressed genes (DEGs) were identified as genes with an adjusted *p* value < 0.05 and |log_2_ (Fold change)| > 1. Functional enrichment analyses for DEGs, encompassing both Gene Ontology (GO) and Kyoto Encyclopedia of Genes and Genomes (KEGG) pathways, were conducted utilizing the clusterProfiler R package (version 4.4.4) within the R software environment (version 4.0). Specifically, GO analysis incorporated the Wallenius non-central hypergeometric distribution model to minimize bias, whereas KEGG analysis was performed in conjunction with the KOBAS database.

### 2.10. Statistical Analysis

The growth parameters were calculated using the following formula:

WGR (%) = (FW − IW) × 100%/IW(1)

SGR (%/d) = ln (FW/IW) × 100%/t(2)

FCR = TI/(FW − IW)(3)

In the above formulas, WGR, SGR, and FCR represent the weight gain rate (%), specific growth rate (%/d), and feed conversion ratio, respectively. IW and FW represent the initial and final body weight (g), t represents the experimental duration (d), and TI represents the total dry matter intake (g).

All data are presented as mean ± standard error of the mean (SEM). Prior to statistical analysis, normality and homogeneity of variance were assessed for each parameter across experimental groups using SPSS 27.0 (Chicago, IL, USA). When assumptions of normality and homoscedasticity were satisfied, one-way analysis of variance (ANOVA) was performed, followed by Tukey’s test for pairwise multiple comparison. Statistical significance was set at *p* < 0.05. Graphical representations were generated using GraphPad Prism 10.4 (San Diego, CA, USA).

## 3. Results

### 3.1. Growth Performance

**Figure 1:**
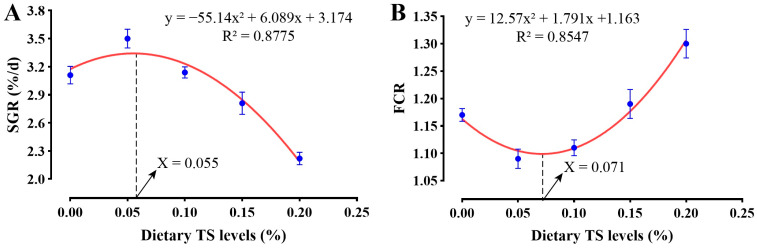
Fitting curves of specific growth rate (SGR, **A**) and feed conversion ratio (FCR, **B**) against dietary TS levels in hybrid grouper fed high-lipid, low-protein diets.

As shown in Table 3, dietary supplementation with TS influenced growth performance parameters in hybrid grouper. Compared with the control group (T0), only the T5 group exhibited significantly higher FAW (*p* < 0.05). Regarding WGR, the T5 group was numerically higher but not statistically different from the T0 group. No significant differences in FAW or WGR were observed between the T10 or T15 groups and T0, and T20 was significantly lower than T0 group. SGR did not differ significantly between the T5 and T0 groups (*p* > 0.05). However, TS supplementation exceeding 0.10% reduced growth performance across all groups, with the lowest values in T20. Second-order polynomial regression analysis of SGR and FCR against dietary TS levels (Figure 1) revealed quadratic relationships. The SGR curve increased initially, peaking at a TS inclusion level of 0.055%, and declined at concentrations above this level. Conversely, the FCR curve showed a declining trend initially and reached a minimum value at 0.071%. Based on these models, the optimal dietary TS supplementation levels for maximizing growth and feed efficiency in hybrid grouper fed a low-protein, high-lipid diet are estimated to be 0.055% and 0.071%, respectively.

### 3.2. Histomorphology of the Liver

**Figure 2:**
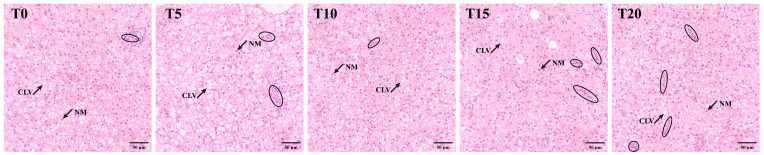
Liver histomorphology of groupers fed high-lipid, low-protein diets with added TS (H&E staining; Scale bar = 50 μm). NM: nucleus migration; CLV: cell lipid vacuolation. The circles or ovals indicate infiltration of inflammatory cells.

Histological images of hepatic tissue from groupers fed different diets supplemented with varying levels of TS are shown in Figure 2. The T10 group appeared to have reduced inflammatory infiltration compared to the other groups. Hepatocytes in the T10 group were more uniform in size and morphology and displayed better cellular alignment. In the T15 group, lipid-type vacuolation was less pronounced than in the control, suggesting partial amelioration of hepatic steatosis; however, hepatocyte boundaries became indistinct, indicating potential cellular damage or swelling.

### 3.3. Antioxidant Capacity and Cellular Stress Response

**Figure 3:**
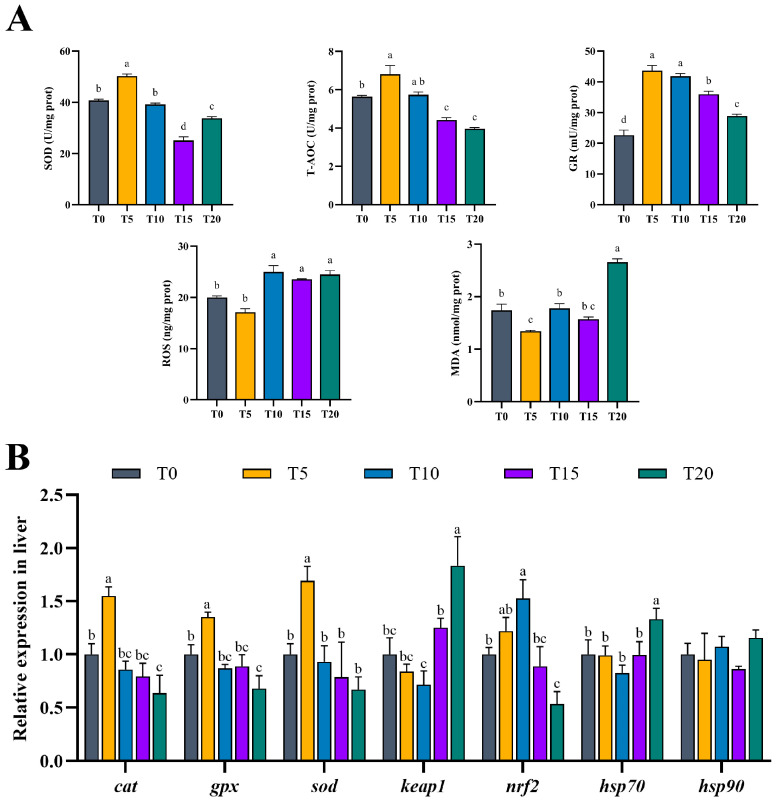
Effects of TS supplementation in high-lipid, low-protein diets on hepatic antioxidant parameters and stress-related gene expression. (**A**) Activities of superoxide dismutase (SOD), glutathione reductase (GR), and total antioxidant capacity (T-AOC), as well as the contents of reactive oxygen species (ROS) and malondialdehyde (MDA). (**B**) Relative mRNA expression levels of antioxidant-related genes (*cat*, catalase; *gpx*, glutathione peroxidase; *sod,* superoxide dismutase; *keap1*, kelch-like epichlorohydrin associated protein 1; *nrf2*, nuclear factor E2related factor 2) and cellular stress response (*hsp70*, heat shock protein 70; *hsp90*, heat shock protein 90). Values are means ± SEM (n = 3). Significant variation in means among the experimental diet groups was indicated by the different lowercase letters (*p* < 0.05).

As shown in Figure 3A, dietary supplementation with TS modulated hepatic antioxidant enzyme activities and biochemical parameters in hybrid grouper. The T5 group exhibited significantly higher SOD activity and T-AOC compared to the control (T0) (*p* < 0.05), and the T15 and T20 groups were significantly lower than the control group (*p* < 0.05). GR levels were significantly higher in TS-supplemented groups compared to T0, but decreased progressively with increasing TS dose (*p* < 0.05). In contrast to antioxidant enzymes, MDA and ROS levels were significantly reduced in T5 compared to T0 (*p* < 0.05), whereas all other groups were significantly higher than the T5 group (*p* < 0.05).

Regarding gene expression (Figure 3B), compared with the control group, the T5 group exhibited significantly higher expression of the antioxidant genes *cat*, *gpx*, and *sod* (*p* < 0.05). However, the expression of these genes decreased progressively with increasing TS levels. Although the T10 and T15 groups showed lower expression than T0, the differences were not statistically significant (*p* > 0.05). For the related signaling pathway, the expression of *nrf2* was higher in T5 compared to T0, though without statistical significance (*p* > 0.05). Notably, *nrf2* expression peaked in the T10 group and was significantly higher than that in T0 (*p* < 0.05). In parallel, *keap1* exhibited lower expression in the T5 and T10 groups compared to T0. Additionally, in terms of stress-related genes, *hsp70* expression was significantly upregulated in the T20 group compared to T0 (*p* < 0.05). In contrast, no significant differences in *hsp90* expression were observed among all groups (*p* > 0.05); nevertheless, both *hsp70* and *hsp90* reached their highest expression levels in the T20 group.

### 3.4. Immune Parameters and Inflammatory Cytokine Expression

**Figure 4:**
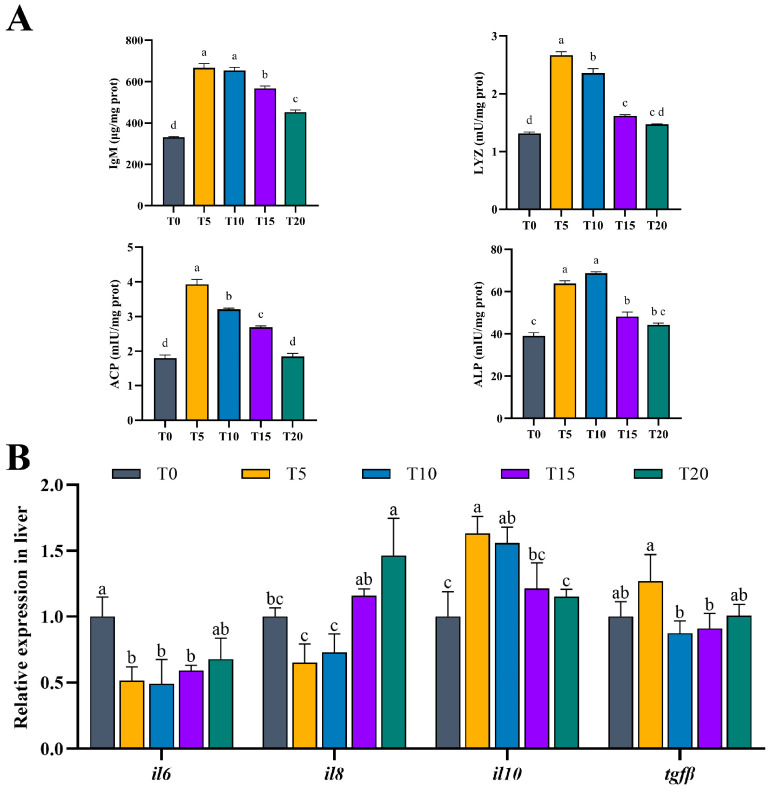
Effects of TS supplementation in high-lipid, low-protein diets on hepatic immune parameters and inflammatory cytokine gene expression. (**A**) Content of immunoglobulin M (IgM) and activities of acid phosphatase (ACP), lysozyme (LYZ), and alkaline phosphatase (ALP). (**B**) Relative mRNA expression levels of pro-inflammatory cytokines (*il6*, interleukin 6; *il8*, interleukin 8) and anti-inflammatory cytokines (*il10*, interleukin 10; *tgfβ*, transforming growth factor beta). Values are means ± SEM (n = 3). Significant variation in means among the experimental diet groups was indicated by the different lowercase letters (*p* < 0.05).

As presented in Figure 4A, IgM, LYZ, ACP and ALP activities were significantly higher in the T5–T15 groups compared to the T0 group (*p* < 0.05), and these immunological indicators showed a significant decreasing trend with increasing additive dosage (*p* < 0.05).

Regarding inflammatory cytokine genes (Figure 4B), the expression of anti-inflammatory cytokines showed distinct patterns. *il10* expression was significantly elevated in both T5 and T10 groups relative to T0 (*p* < 0.05). The expression of *tgfβ* was also higher in T5 compared to T0, though without statistical significance (*p* > 0.05). Conversely, the pro-inflammatory cytokines (*il6* and *il8*) exhibited lower expression in the T5 and T10 groups compared to T0. Overall, as dietary TS supplementation increased from 0% to 0.20%, the expression of these pro-inflammatory cytokines initially declined (reaching minima at T5 or T10), and then increased in the higher-dose groups (T15 and T20).

### 3.5. Hepatic Transcriptome Analysis

#### 3.5.1. Sequencing Quality Assessment and Reference Genome Mapping

Transcriptome sequencing was conducted using three biological replicates per group, yielding a total of 58.89 Gb of clean data. After quality filtering, 197,639,856 high-quality clean reads were obtained for downstream analyses. The GC content of all samples ranged from 47.27% to 49.13%. Moreover, the sequencing quality was high, with Q20 values ranging from 97.65% to 97.92% and Q30 values ranging from 94.13% to 94.82%. All clean reads from the nine samples were aligned to the *Epinephelus lanceolatus* reference genome, the paternal species of the hybrid grouper, resulting in an average mapping rate of 71.19%. The number of mapped reads and the corresponding mapping ratios for each sample are presented in Table 4.

#### 3.5.2. Principal Component Analysis and Differential Expression Statistics

**Figure 5:**
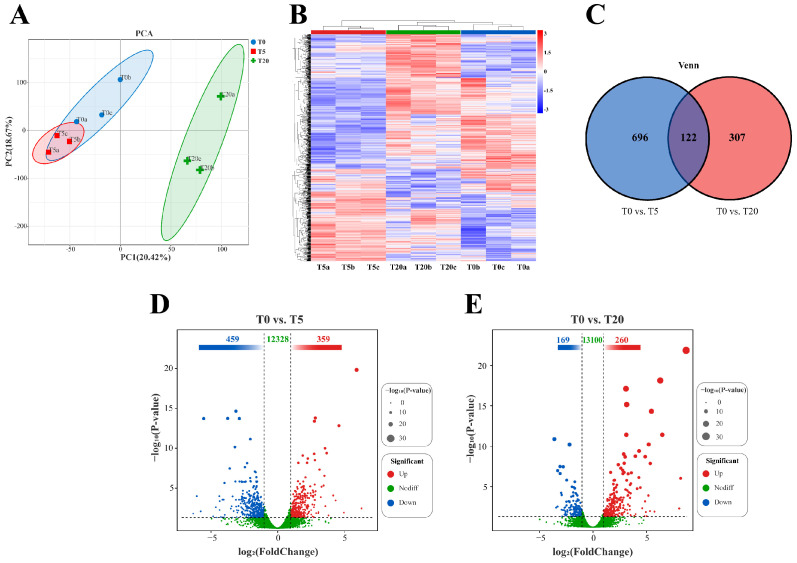
Sample relationships and differentially expressed gene (DEG) profiles in grouper fed diets with different TS intakes. (**A**) Principal component analysis (PCA) of hepatic gene expression. (**B**) Hierarchical clustering heatmap of hepatic DEGs. (**C**) Venn diagram of DEGs among comparisons; the sum of numbers within each circle indicates the total number of DEGs in that comparison, and the overlapping regions represent DEGs shared between comparisons. (**D**,**E**) Volcano plots of DEGs for each comparison. Significantly upregulated genes are shown in red, and significantly downregulated genes are shown in blue.

Principal component analysis (PCA) of the transcriptomes from the T0, T5, and T20 groups (Figure 5A) showed that principal component 1 (PC1) and principal component 2 (PC2) explained 20.42% and 18.67% of the total variance, respectively. Samples within the T5 group clustered tightly. Upon the initiation of TS supplementation, the T5 group shifted away from the T0 group, whereas a further increase in TS intake resulted in a clear separation between the T20 and T5 groups. Overall, all samples were distinctly separated into three clusters, indicating that different TS doses markedly affected hepatic gene expression in grouper. Comparisons were conducted for T0 vs. T5 and T0 vs. T20. Hierarchical clustering and heatmap visualization based on DEG expression (Figure 5B) further demonstrated distinct expression patterns under different TS intakes. As shown in Figure 5C, only 122 DEGs were shared among the two comparisons. The T0 vs. T5 comparison yielded the largest number of DEGs (818). Compared with the T0 group, the T5 group showed 359 upregulated and 459 downregulated genes, whereas the T20 group showed 260 upregulated and 169 downregulated genes (Figure 5D,E).

#### 3.5.3. GO Annotation and Functional Classification

**Figure 6:**
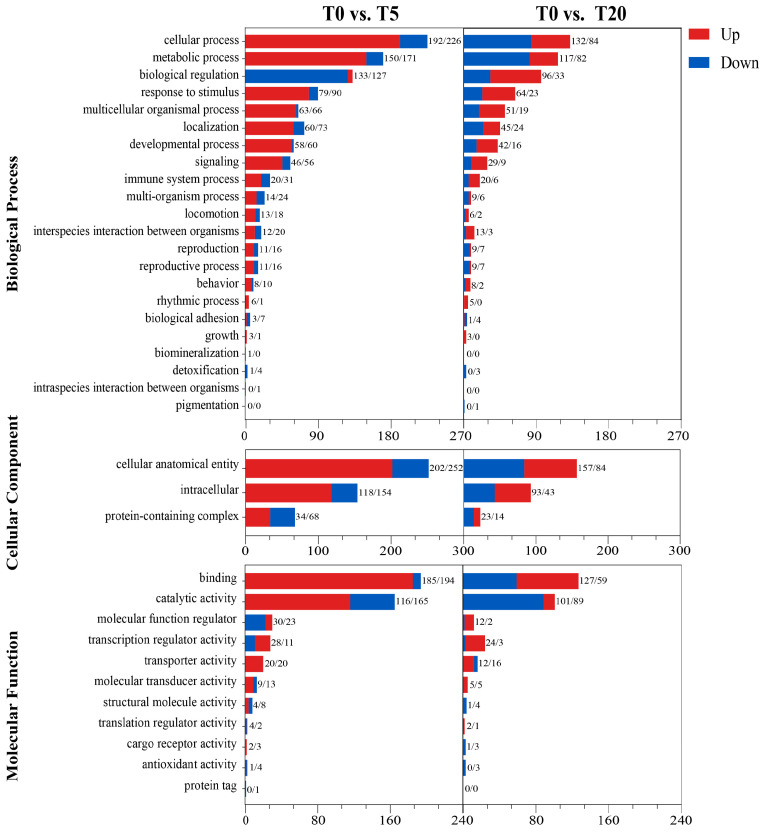
GO functional classification of DEGs under different TS supplementation levels.

As shown in Figure 6, DEGs were primarily enriched in the three major GO level 2 categories: biological process (BP), cellular component (CC), and molecular function (MF). Within BP, most DEGs were assigned to cellular process, metabolic process, and biological regulation. Notably, the T0 vs. T5 group showed a higher number of DEGs in immune- and inflammation-related terms, including response to stimulus, signaling, and immune system process, whereas the overall number of DEGs annotated to these immune-related terms was lower in T0 vs. T20 than in T0 vs. T5. In CC, DEGs were mainly associated with cellular anatomical entity and intracellular. In MF, binding and catalytic activity were the most represented subcategories. In addition, no upregulated genes were annotated to antioxidant activity in the T0 vs. T20 group.

#### 3.5.4. KEGG Annotation Classification and Enrichment Analysis

**Figure 7:**
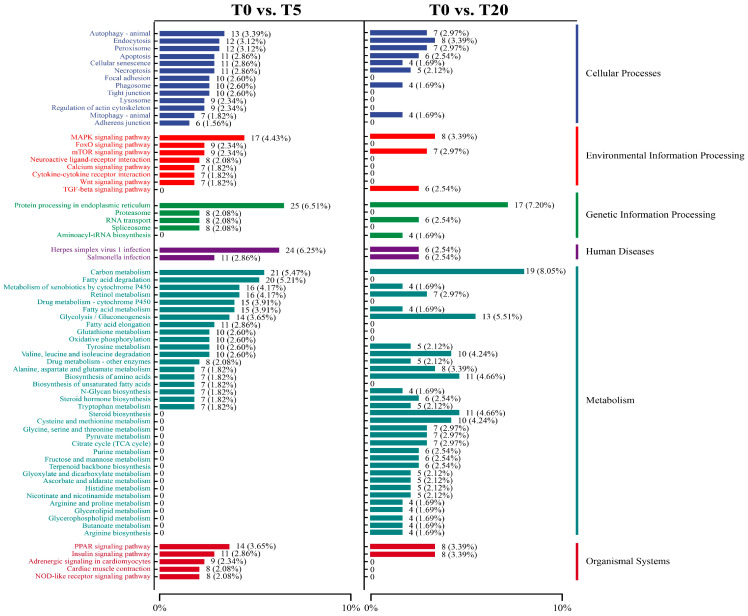
KEGG functional classification of DEGs under different TS supplementation levels.

KEGG functional classification (Figure 7) showed that, compared with the T0 group, the pathway distribution in the T5 group was more prominently oriented toward inflammation-regulatory networks. Within the “cellular processes” category, the T0 vs. T5 comparison exhibited higher proportions of pathways related to barrier homeostasis, including autophagy, lysosome, phagosome, endocytosis, as well as tight junction, adherens junction, and regulation of the actin cytoskeleton. In contrast, DEGs in the T0 vs. T20 comparison were more strongly associated with fundamental metabolic processes and protein homeostasis, including carbon metabolism, glycolysis/gluconeogenesis, amino acid metabolism, and protein processing in the endoplasmic reticulum.

**Figure 8:**
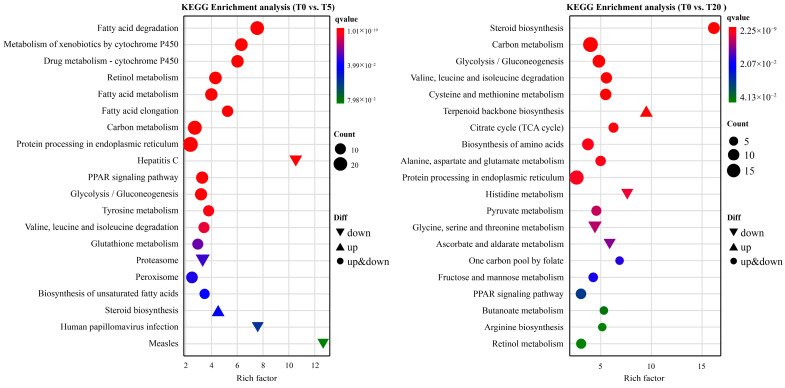
KEGG pathway enrichment of DEGs under different TS supplementation levels (bubble plot). Bubble size represents the number of genes enriched in each pathway, while color intensity indicates statistical significance (*p*-value). The rich factor is calculated as the ratio of differentially expressed genes to the total annotated genes within the pathways.

KEGG pathway enrichment analysis showed that DEGs in T5 were predominantly enriched in fatty acid degradation, fatty acid metabolism, fatty acid elongation, and PPAR signaling pathway relative to T0 (Figure 8). Additionally, pathways related to oxidative stress buffering and inflammation regulation were enriched, including peroxisome, glutathione metabolism, and proteasome. In the T0 vs. T20 comparison, enriched pathways were mainly related to steroid biosynthesis and central carbon metabolism-related pathways, accompanied by downregulation of histidine metabolism and ascorbate and aldarate metabolism.

#### 3.5.5. Gene Expression Trend Analysis and Screening of Immune-Related Genes

**Figure 9:**
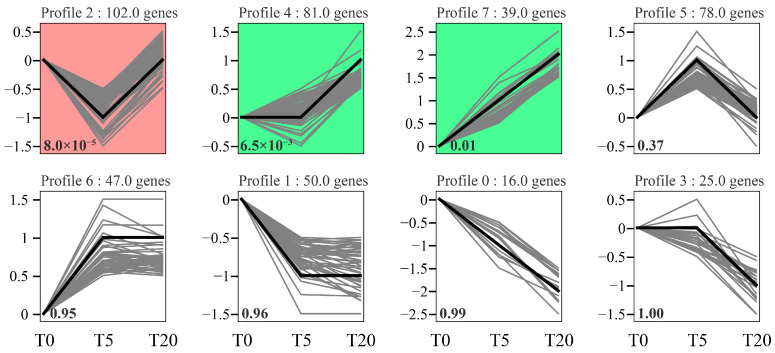
Eight expression patterns of the DEGs. The colored backgrounds for Profile 2, Profile 4, and Profile 7 indicate significant trends (*p* < 0.05). with modules showing similar expression patterns displayed in the same color. The curves reflect changes in gene expression with increasing additive dosage.

The expression profiles of all co-expressed genes were classified into 8 distinct patterns, of which three exhibited statistical significance (*p* < 0.05), encompassing 102, 81, and 39 genes in Profiles 2, 4, and 7, respectively (Figure 9). Profile 2 displayed a V-shaped pattern, where gene expression declined at the T5 dose but rebounded at the T20 dose. By contrast, Profile 4 remained relatively stable from T0 to T5 and then increased toward T20, whereas Profile 7 showed a progressive increase from T0 to T20. Further screening identified a subset of immune-related genes within these significant profiles (Table 5). Within Profile 2, inflammation and ER stress markers (*il1rl1*, *map3k14a*, *psmb9a*, *eif2ak2*, *noxo1a*) and ER chaperones (*hsp90b1*, *calr*, *pdia3*) were suppressed in T5 but upregulated in T20. Profiles 4 and 7, however, exhibited progressive upregulation of genes involved in autophagy (*vmp1*, *tfe3a*) and inflammatory signaling (*tlr5*, *atf3*, *ddit3*).

## 4. Discussion

TS, the primary bioactive triterpenoid saponin derived from camellia seed meal, has garnered attention in aquaculture due to its potential growth-promoting and immunomodulatory properties. Although earlier studies did not conclusively demonstrate that purified TS enhances fish growth, dietary inclusion of camellia seed meal—containing low concentrations of TS—was reported to improve growth performance, protein utilization, and non-specific immunity in *Oreochromis niloticus* [39] and Gibel carp (*Carassius auratus gibelio*) [40]. These benefits were attributed to the latent bioactivity of low-dose TS.

In the present study, dietary supplementation with 0.05% TS in a high-lipid, low-protein diet improved growth performance, with a significant increase observed in FAW and reduced feed conversion ratio (FCR) compared with the control (T0), whereas higher supplementation levels (≥0.15%) impaired growth. This biphasic response aligns with findings in other fish species fed triterpenoid saponins. For instance, 0.16% *Momordica charantia* saponins significantly enhanced WGR and SGR in common carp fed a low-protein, high-carbohydrate diet [41], and 0.03% Quillaja saponin increased body weight and reduced FCR in *Oreochromis niloticus* [42]. Despite differences in experimental conditions and fish species, growth-promoting effects of triterpenoid saponins have been consistently observed at appropriate supplementation levels. Conversely, 0.2% soya saponins reduced growth in juvenile turbot (*Scophthalmus maximus*) fed a fishmeal-based diet [43], while 0.08% soya saponins promoted growth but 0.64% suppressed it in Japanese flounder (*Paralichthys olivaceus*) [44]. Collectively, these studies support a hormetic effect of saponins: beneficial at low doses but inhibitory or toxic at high doses [45].

The growth enhancement observed with 0.05% TS may be related to a partial relief of the metabolic burden imposed by the low-protein, high-lipid diet. In fish, excessive lipid can suppress PPARα-related fatty-acid transport and β-oxidation and trigger ER stress-associated defects in VLDL export, thereby promoting triglyceride retention, oxidative stress, inflammation and apoptosis [46,47]. Such disturbances may further impair hepatointestinal lipid trafficking and nutrient partitioning, lowering the efficiency with which dietary energy is converted into somatic growth, while chronic cellular stress diverts nutrients from growth toward maintenance and defense. Meanwhile, reduced protein and essential amino acid supply can restrain PI3K/AKT/TOR-S6K1 signaling, muscle protein deposition, and the synthesis of barrier and immune effectors required for intestinal integrity and metabolic homeostasis [48,49,50], suggesting that the modest growth advantage in the T5 group was associated with a more balanced use of dietary lipid and protein under this nutritionally challenging condition. In contrast, the elevated FCR in the T20 group likely reflects reduced feed intake caused by the bitter taste of TS [51], consistent with reports that various botanical saponins suppress appetite and nutrient absorption [52]. Second-order polynomial regression of SGR further confirmed an optimal TS inclusion level of 0.055%, beyond which growth declined sharply.

High-lipid diets are known to induce hepatic oxidative stress, inflammatory responses, and structural damage [22,53,54], whereas protein deficiency impairs tissue integrity and antimicrobial synthesis [55,56]. In our study, supplementation with 0.05% TS ameliorated hepatic morphology, as evidenced by hepatocytes of uniform size, tighter cellular alignment, and reduced inflammatory infiltration, collectively reflecting a marked attenuation of hepatic injury. Although mild vacuolation was observed in the T5 group, this likely reflects transient lipid accumulation rather than pathological steatosis. By contrast, T15 and T20 groups displayed indistinct hepatocyte boundaries, cellular fragmentation, and pronounced inflammation, suggesting dose-dependent hepatotoxicity.

Biochemically, T5 significantly increased T-AOC and activities of SOD and GR, while reducing MDA and ROS levels (*p* < 0.05). These findings corroborate studies in grass carp [57], largemouth bass (*Micropterus salmoides*) [58], and snakehead (*Channa argus*) [59], where optimal micronutrient or antioxidant supplementation enhanced T-AOC and suppressed MDA, whereas excess doses reversed these effects. The coordinated upregulation of *sod*, *cat*, and *gpx* in T5 further supports enhanced enzymatic ROS scavenging.

However, unlike the antioxidant enhancement commonly reported under moderate phytochemical supplementation, at TS ≥ 0.10%, antioxidant gene expression and enzyme activities declined, despite elevated GR activity in T15 and T20. Notably, ROS levels did not increase further in T15/T20, possibly due to direct free radical scavenging by high-dose TS via non-enzymatic pathways [60,61]. Nevertheless, T-AOC was markedly reduced in T15 and T20 (*p* < 0.05), indicating compromised overall antioxidant defense. The dissociation between GR activity and T-AOC suggests a functional impairment of the GSH-dependent antioxidant system, rather than an overall enhancement of redox homeostasis. One plausible explanation is inadequate GSH regeneration capacity, as *gpx* expression was not concomitantly upregulated, limiting H_2_O_2_ clearance [62,63]. Furthermore, the progressive downregulation of *sod* and *cat* suggests impaired superoxide dismutation and subsequent H_2_O_2_ detoxification, which may constrain substrate availability for the GSH/GR pathway. As the liver is the main site of GSH synthesis and a major determinant of T-AOC, disruption of hepatic GSH utilization and recycling under high TS exposure may explain the reduced antioxidant capacity, despite elevated GR activity. Excessive TS supplementation may induce oxidative damage and apoptosis. These observations parallel reports of hepatotoxicity from high-dose triterpenoid saponins in mammals [64,65], underscoring the narrow therapeutic window of phytochemicals like TS.

Dietary saponins are well-established immunostimulants in aquaculture, known to modulate innate immunity through enhancing phagocytosis, stimulating antibody production, and antimicrobial activity in aquatic animals [66,67]. In the present study, the immunosuppressive effects typically associated with low-protein, high-lipid diets, including reduced immune competence and enzymatic defenses [68,69], were alleviated by TS supplementation. At 0.05% TS significantly increased hepatic non-specific humoral immune indicators, including IgM, LYZ, ACP, and ALP, compared with the control (*p* < 0.05). This immunostimulatory response is consistent with previous findings on ginseng stem and leaf saponins [67] and yucca saponins [70]. Notably, immune indices peaked at T5 and declined at higher inclusion levels, following a dose-dependent trend similar to that observed in growth and antioxidant parameters and consistent with reports on curcumin supplementation in fish [71].

Critically, TS modulated the balance between pro- and anti-inflammatory cytokines. Expression of pro-inflammatory cytokine genes (*il6*, *il8*) was suppressed in T5 and T10, while anti-inflammatory *il10* was significantly upregulated (*p* < 0.05). In parallel, *keap1*—a negative regulator of *nrf2*—was also downregulated in T5 and T10, which may facilitate *nrf2* activation and strengthen antioxidant defenses. Although *tgfβ* and *nrf2* showed non-significant increases in T5, *nrf2* peaked in T10 and was significantly higher than T0. Morphologically, reduced inflammatory cell infiltration in T10 further supports an anti-inflammatory effect of moderate TS supplementation.

The Nrf2–Keap1 pathway plays a central role in redox homeostasis. Under oxidative stress, Nrf2 dissociates from Keap1, translocates to the nucleus, and activates antioxidant genes. In our study, hepatic *nrf2* expression peaked at 0.10% TS but declined sharply at 0.20%, while *keap1* increased at ≥0.15%. This suggests that excessive TS disrupts the Nrf2–Keap1 axis, impairing the adaptive antioxidant response and exacerbating oxidative injury.

To further elucidate the molecular underpinning of the biphasic immunomodulatory and hepatoprotective effects of TS, we performed hepatic transcriptomic profiling in hybrid grouper fed a low-protein, high-lipid diet supplemented with 0% (T0), 0.05% (T5), or 0.20% (T20) TS. PCA and DEG-based clustering revealed a clear, non-linear dose-dependent divergence in hepatic transcriptional landscapes. Notably, the T5 group exhibited the most extensive remodeling to T0, while only a limited set of DEGs were shared across all pairwise comparisons—strongly supporting a hormetic response pattern, wherein low-dose exposure elicits adaptive benefits that are lost or reversed at higher doses [72]. This non-monotonicity underscores that TS does not act as a simple linear modulator but, rather, engages distinct biological networks depending on concentration. KEGG functional classification and enrichment analyses provided critical insights into the nature of these dose-specific programs. In the T5 group, pathway enrichment was strikingly oriented toward inflammation-containment, redox homeostasis, and cellular quality control. Specifically, T5 exhibited elevated representation of autophagy, lysosome, phagosome/endocytosis, and cytoskeleton/junction-associated pathways. These pathways are increasingly recognized as central to maintaining hepatic integrity in teleosts: they facilitate the clearance of intracellular pathogens and damaged organelles, regulate vesicular trafficking to limit danger-associated molecular pattern (DAMP) release, and preserve intercellular barrier function to prevent systemic inflammation [73]. The coordinated upregulation of such pathways aligns with our histological observations of reduced inflammatory infiltration and preserved hepatocyte architecture in T5.

Concurrently, T5 exhibited significant enrichment in peroxisome, glutathione metabolism, and proteasome pathways—a triad that collectively enhances cellular capacity for ROS detoxification and protein turnover. Peroxisomes generate H_2_O_2_ as a byproduct of fatty acid β-oxidation but also house catalase for its neutralization; their expansion often reflects adaptive metabolic rewiring under lipotoxic stress. Glutathione metabolism provides the primary reducing equivalent for antioxidant genes like *gpx*, while the ubiquitin–proteasome system degrades oxidized or misfolded proteins. This integrated defense network corroborates our biochemical data and gene expression data showing elevated T-AOC, SOD activities and *cat*, *gpx*, and *sod* expression in T5 liver tissue, confirming that low-dose TS fortifies both enzymatic and non-enzymatic antioxidant systems via transcriptional activation.

To link pathway-level enrichment with a mechanistic dose–response framework, we analyzed the expression trajectories of immune- and stress-related DEGs across the TS gradient. Three statistically significant patterns emerged, a V-shaped pattern (Profile 2), a progressively increasing pattern (Profile 7), and a late-response pattern (Profile 4). These trajectories help to explain the biphasic phenotype, in which low-dose TS improved immune status and alleviated inflammation, whereas excessive TS reversed these benefits. V-shaped pattern genes included regulators linked to inflammatory signaling and immune activation (*map3k14a*, *il1rl1*, *psmb9a*), interferon/stress-associated factors (*eif2ak2*, *ifi44l*), and a suite of ER folding/quality-control genes (e.g., *hsp90b1*, *calr*/*canx*, and *pdia* family members). Their suppression at T5 aligns with higher *il10* and lower *il6*/*il8* in T5, together with enhanced humoral immune indices (IgM, LYZ, ACP, ALP), which peaked at T5 and declined with increasing TS. Conversely, re-activation of these modules at high TS agrees with reduced T-AOC/SOD and aggravated histological injury in T20, as well as the marked induction of *hsp70* in T20, indicating a shift toward stress-associated inflammation.

The toxicity mechanism at high doses is further supported by the progressively increasing and late-response clusters. The monotonic upregulation of *tlr5* and AP-1 family members (*junbb*, *fosl1a*, *fosl2*) suggests sustained innate immune activation [74,75]. Meanwhile, the induction of the integrated stress response effectors *ddit3* (CHOP) and *chac1* indicates maladaptive ER stress, with CHAC1-mediated glutathione degradation likely weakening cellular redox defense [76]. Given that membrane-active saponins interact strongly with cholesterol, reduce membrane cholesterol, and lose much of their lytic activity after cholesterol depletion [77,78,79], excessive TS in T20 may have directly destabilized hepatocyte membranes and increased membrane permeability, thereby contributing to hepatocyte rupture and blurred cellular boundaries. This inference is supported by aquatic evidence showing that dietary soya saponins are associated with hepatocyte atrophy in rainbow trout and, at higher levels, organelle injury, ER dilation, elevated ALT/AST and total bile acid, and impaired antioxidant capacity in largemouth bass [80,81]. In our study, such primary membrane injury likely converged with *tlr5*/AP-1 activation and *ddit3*/*chac1* induction, translating structural damage into inflammatory rebound and glutathione-consuming ER stress, whereas the late upregulation of *vmp1*, *tfe3a*, and *manf* more likely reflects secondary rescue than homeostatic adaptation. Furthermore, unlike the homeostatic autophagy observed in T5, the late induction of autophagy/lysosome regulators (*tfe3a*, *vmp1*) and cytoprotective factors (*manf*) in T20 likely reflects a secondary compensatory response to alleviate severe cellular damage [82].

Collectively, our integrated physiological, molecular, and transcriptomic data demonstrate that dietary supplementation with TS at 0.05–0.10% effectively counteracts the detrimental effects of low-protein, high-lipid diets in hybrid grouper through a multi-faceted protective mechanism: it could enhance growth performance by improving nutrient utilization, alleviating hepatic oxidative stress through Nrf2-mediated transcriptional activation of antioxidant genes and modulating immune homeostasis via suppression of pro-inflammatory cytokines and upregulation of humoral immune factors. Transcriptome analysis further revealed that these benefits arise from the coordinated activation of PPAR signaling, fatty acid catabolism, autophagy–lysosome flux, and redox-buffering systems (e.g., glutathione metabolism and peroxisome pathways), collectively forming a robust, multi-layered defense against diet-induced metabolic and inflammatory stress. In stark contrast, TS concentrations at or above 0.15% induce hepatotoxicity, characterized by oxidative damage, unresolved inflammation, and metabolic dysfunction. These adverse effects are likely driven by excessive ROS generation, suppression of the Nrf2–Keap1 antioxidant axis as evidenced by declining nuclear factor erythroid 2-related factor 2 (*nrf2*) and rising Kelch-like ECH-associated protein 1 (*keap1*) expression at high doses, and direct membrane disruption by saponin and cholesterol interactions [64,65,83]. Based on quadratic regression modeling of SGR, the optimal dietary TS level under these nutritional conditions is approximately 0.055%, representing a narrow but critical therapeutic window that maximizes growth, antioxidant capacity, and immune function without eliciting cytotoxic or inflammatory adverse effects. Future studies will explore how tea saponin modulates gut microbiota composition and bile acid metabolism to further elucidate the underlying mechanisms governing its systemic effects in carnivorous fish.

## 5. Conclusions

This study demonstrates that dietary TS supplementation exerts dose-dependent effects on hybrid grouper fed high-lipid, low-protein diets. Within the optimal range of 0.05–0.10%, TS effectively mitigated the adverse consequences of nutritional imbalance by improving growth performance, alleviating hepatic oxidative stress, and reinforcing immune homeostasis, with these benefits coordinated through the transcriptional activation of lipid metabolism, autophagy–lysosome, and redox-buffering pathways. Conversely, supplementation at or above 0.15% induced pronounced hepatotoxicity and systemic dysregulation, indicating that TS exerts beneficial effects only within a narrow therapeutic window. Strict dosage control is therefore essential; feed manufacturers should ensure TS inclusion does not exceed 0.10% in hybrid grouper diets. Based on quadratic regression of SGR and FCR, the estimated optimal TS levels for maximizing growth and feed efficiency are 0.055% and 0.071%, respectively. Whether these effects extend to other carnivorous fish species under similar nutritional stress warrants further investigation. The present study was conducted over a relatively short 4-week period under controlled laboratory conditions, which may not fully capture the long-term growth performance observed in commercial aquaculture settings. Future studies should therefore extend the trial duration under field conditions and incorporate established additives, such as vitamin E or β-glucan, as reference benchmarks to better validate and contextualize these findings.

## Figures and Tables

**Table 1:** Formulation and proximate composition (dry matter basis, %).

Ingredient (%)	Groups				
	T0	T5	T10	T15	T20
Fish meal	30.00	30.00	30.00	30.00	30.00
Soybean meal	8.00	8.00	8.00	8.00	8.00
Clostridium autoethanogenum protein	17.00	17.00	17.00	17.00	17.00
Wheat flour	18.00	18.00	18.00	18.00	18.00
Pregelatinized starch	3.00	3.00	3.00	3.00	3.00
Phospholipid	1.50	1.50	1.50	1.50	1.50
Fish oil	5.00	5.00	5.00	5.00	5.00
Corn oil	7.00	7.00	7.00	7.00	7.00
Premix ^a^	1.00	1.00	1.00	1.00	1.00
Vitamin C	0.05	0.05	0.05	0.05	0.05
Monocalcium phosphate	1.50	1.50	1.50	1.50	1.50
Antioxidant	0.05	0.05	0.05	0.05	0.05
Attractant	0.20	0.20	0.20	0.20	0.20
Choline chloride	0.50	0.50	0.50	0.50	0.50
Microcrystalline cellulose	7.20	7.15	7.10	7.05	7.00
Tea saponin	0.00	0.05	0.10	0.15	0.20
Total	100.00	100.00	100.00	100.00	100.00
Proximate composition ^b^ (%)					
Crude protein	42.47	42.02	42.31	42.77	42.23
Crude lipid	16.07	16.22	15.90	16.36	16.43

Notes: Nutrient ratios of the main ingredients in the feed (dry matter basis): Fish meal: Crude fat, 6.31%, Crude protein, 68.36%; Soybean meal: Crude fat, 1.01%, Crude protein, 52.56%; Clostridium autoethanogenum protein: Crude fat, 0.32%, Crude protein, 88.48%; Wheat flour: Crude fat, 1.39%, Crude protein, 14.28%. ^a^ Premix provided by Qingdao Master Biotechnology Co., Ltd. (Qingdao, China). ^b^ The actual values that were measured for proximate nutritional composition (dry matter basis).

**Table 2:** Primer sequences of target genes used for RT-qPCR analysis.

Primer Name	Forward Primer (5′-3′)	Reverse Primer (5′-3′)	Accession No.
*cat*	GCAAGTTCCACTACAAGACTG	GCATAATCTGGGTTGCTGGA	XM_049573567.1
*gpx*	TCCTCTGTGGAAGTGGCTGA	TCATCCAGGGGTCCGTATCT	XM_033622197.1
*sod*	CAGTGGGACCGTGTATTTTGAG	CAGTCACATTTCCCAGGTCTCC	XM_049593128.1
*hsp70*	ATCAATCCAGACGAGGCA	TACCCAGGGACAGAGGC	XM_049561556.1
*hsp90*	AACGACAAGGCTGTGAAGGAC	TTCTGTAGATGCGGTTGGAGTG	XM_049590777.1
*il6*	CAATCCCAGCACCTTCCAC	CCTGACAGCCAGACTTCCTCT	XM_049603149.1
*il8*	TGTGGCACTCCTGGTTCTCC	GGGTTCACCTCCACCTGTCC	XM_049572727.1
*keap1*	TCCACAAACCCACCAAAGTAA	TCCACCAACAGCGTAGAAAAG	XM_033623805.1
*il10*	CGGAGTGACGGAGGATACCA	AACCTTTACCCTCCATCTGAGT	XM_049580695.1
*tgfβ*	CCGCTTCATCACCAACGAG	CCGCTCATCCTCATTTCCTT	XM_049576571.1
*nrf2*	TATGGAGATGGGTCCTTTGGTG	GCTTCTTTTCCTGCGTCTGTTG	XM_033617942.1
*β-actin*	GGCTACTCCTTCACCACCACA	TCTGGGCAACGGAACCTCT	XM_033645256.1

Note: *cat* (catalase); *gpx* (glutathione peroxidase); *sod* (superoxide dismutase); *hsp70* (heat shock protein 70); *hsp90* (heat shock protein 90); *il6* (interleukin 6); *il8* (interleukin 8); *keap1* (kelch-like epichlorohydrin associated protein 1); *il10* (interleukin 10); *tgfβ* (transforming growth factor beta); *nrf2* (nuclear factor E2related factor 2); *β-actin* (beta actin).

**Table 3:** Growth parameters of groupers fed high-lipid, low-protein diets with added TS.

Parameters	Groups				
	T0	T5	T10	T15	T20
IAW (g)	17.48 ± 0.01	17.53 ± 0.00	17.54 ± 0.02	17.52 ± 0.02	17.50 ± 0.02
FAW (g)	41.76 ± 1.05 ^b^	46.70 ± 1.33 ^a^	42.25 ± 0.71 ^ab^	38.55 ± 1.25 ^b^	32.56 ± 0.58 ^c^
WGR (%)	138.87 ± 6.14 ^ab^	166.38 ± 7.63 ^a^	140.97 ± 4.09 ^ab^	120.11 ± 7.24 ^b^	86.10 ± 3.39 ^c^
SGR (%/d)	3.11 ± 0.09 ^ab^	3.50 ± 0.10 ^a^	3.14 ± 0.06 ^ab^	2.81 ± 0.12 ^b^	2.22 ± 0.07 ^c^
FCR (%)	1.17 ± 0.01 ^bc^	1.09 ± 0.02 ^c^	1.11 ± 0.01 ^bc^	1.19 ± 0.03 ^b^	1.30 ± 0.03 ^a^

Notes: Figures shown in the table are means ± SEM (n = 3). The same superscript or absence of superscripts in the same row indicates no significant differences (*p* > 0.05). IAW: initial average weight; FAW: final average weight; WGR: weight gain rate; SGR: specific growth rate; FCR: feed conversion ratio.

**Table 4:** Summary statistics of liver transcriptome sequencing data.

Sample	Clean Reads	Clean Bases (bp)	GC Content (%)	Q20 (%)	Q30 (%)	Mapped Reads
T0a	22,378,015	6,666,660,478	48.94	97.74	94.33	33,134,847 (74.03%)
T0b	22,129,577	6,575,286,554	47.27	97.73	94.46	29,369,241 (66.36%)
T0c	21,637,091	6,447,423,232	48.28	97.88	94.70	29,762,760 (68.78%)
T5a	21,601,200	6,428,501,174	49.13	97.92	94.82	31,326,955 (72.51%)
T5b	22,466,820	6,692,240,965	48.85	97.65	94.13	32,924,424 (73.27%)
T5c	21,642,623	6,453,927,172	48.98	97.87	94.70	32,406,611 (74.87%)
T20a	22,344,568	6,671,387,541	47.76	97.70	94.31	30,541,937 (68.34%)
T20b	21,882,123	6,525,852,537	48.39	97.82	94.48	31,463,062 (71.89%)
T20c	21,557,839	6,430,366,288	47.95	97.89	94.76	30,463,912 (70.66%)

**Table 5:** Immune-related differential gene statistics.

Gene ID	Gene Description	T0 vs. T5	T5 vs. T20	T0 vs. T20
117249054	*il1rl1*; interleukin-1 receptor type 1	down	up	normal
117268475	*map3k14a*; mitogen-activated protein kinase kinase kinase 14a	down	up	normal
117263212	*psmb9a*; proteasome 20S subunit beta 9a	down	up	normal
117268736	*jmjd8*; jumonji domain containing 8	down	up	normal
117248633	*eif2ak2*; uncharacterized LOC117248633	down	up	normal
117253731	*ifi44l*; interferon-induced protein 44-like	down	up	normal
117261215	*magt1*; magnesium transporter 1	down	up	normal
117251812	*inhbb*; inhibin subunit beta B	down	up	normal
117268097	*noxo1a*; NADPH oxidase organizer 1a	down	up	normal
117272819	*txnb*; thioredoxin b	down	up	normal
117270274	*mpv17*; mitochondrial inner membrane protein MPV17	down	up	normal
117262168	*kras*; v-Ki-ras2 Kirsten rat sarcoma viral oncogene homolog	down	up	normal
117262185	*hsp90b1*; heat shock protein 90, beta (grp94), member 1	down	up	normal
117253366	*calr*; calreticulin	down	up	normal
117260791	*canx*; calnexin	down	up	normal
117262795	*pdia3*; protein disulfide isomerase family A, member 3	down	up	normal
117264692	*pdia4*; protein disulfide isomerase family A, member 4	down	up	normal
117256474	*fkbp11*; FKBP prolyl isomerase 11	down	up	normal
117252654	*sdf2l1*; stromal cell-derived factor 2-like 1	down	up	normal
117252974	polyubiquitin-like	down	up	normal
117269594	*c7b*; complement component C7	normal	up	up
117268525	*gimap7*; GTPase IMAP family member 7	normal	up	up
117258187	*tfe3a*; transcription factor binding to IGHM enhancer 3a	normal	up	up
117246806	*ddit4*; DNA-damage-inducible transcript 4	normal	up	up
117260013	*vmp1*; vacuole membrane protein 1	normal	up	up
117257312	*manf*; mesencephalic astrocyte-derived neurotrophic factor	normal	up	up
117251091	*dnajc3a*; DnaJ (Hsp40) homolog, subfamily C, member 3a	normal	up	up
117262523	*herpud1*; homocysteine-inducible, endoplasmic reticulum stress-inducible, ubiquitin-like domain member 1	normal	up	up
117260672	*sil1*; SIL1 nucleotide exchange factor	normal	up	up
117271251	*pdia6*; protein disulfide isomerase family A, member 6	normal	up	up
117270621	*tlr5*; toll-like receptor 5	up	up	up
117271294	*atf3*; activating transcription factor 3	up	up	up
117267770	*junbb*; JunB proto-oncogene, AP-1 transcription factor subunit b	up	up	up
117251422	*fosl1a*; FOS like 1, AP-1 transcription factor subunit a	up	up	up
117267250	*fosl2*; FOS like 2, AP-1 transcription factor subunit	up	up	up
117256987	*ddit3*; DNA-damage-inducible transcript 3	up	up	up
117270409	*chac1*; ChaC, cation transport regulator homolog 1	up	up	up
117247618	*maff*; v-maf avian musculoaponeurotic fibrosarcoma oncogene homolog F	up	up	up

Note: “Up” and “Down” indicate significant up-regulation and down-regulation, respectively. “Normal” indicates no significant change. Gene IDs correspond to the NCBI database.

## Data Availability

The data that support the findings of this study are available on request from the corresponding author. The data are not publicly available due to privacy or ethical restrictions.
